# Divergent roles of the Wnt/PCP Formin Daam1 in renal ciliogenesis

**DOI:** 10.1371/journal.pone.0221698

**Published:** 2019-08-30

**Authors:** Mark E. Corkins, Vanja Krneta-Stankic, Malgorzata Kloc, Pierre D. McCrea, Andrew B. Gladden, Rachel K. Miller

**Affiliations:** 1 Department of Pediatrics, Pediatric Research Center, UTHealth McGovern Medical School, Houston, Texas, United States of America; 2 MD Anderson Cancer Center UTHealth Graduate School of Biomedical Sciences, Program in Genes and Development, Houston, Texas, United States of America; 3 MD Anderson Cancer Center UTHealth Graduate School of Biomedical Sciences, Program in Genetics & Epigenetics, Houston, Texas, United States of America; 4 Houston Methodist, Research Institute, Houston, Texas, United States of America; 5 Department of Genetics, University of Texas MD Anderson Cancer Center, Houston, Texas, United States of America; 6 MD Anderson Cancer Center UTHealth Graduate School of Biomedical Sciences, Program in Biochemistry & Cell Biology, Houston, Texas, United States of America; University of Massachusetts Medical School, UNITED STATES

## Abstract

Kidneys are composed of numerous ciliated epithelial tubules called nephrons. Each nephron functions to reabsorb nutrients and concentrate waste products into urine. Defects in primary cilia are associated with abnormal formation of nephrons and cyst formation in a wide range of kidney disorders. Previous work in *Xenopus laevis* and zebrafish embryos established that loss of components that make up the Wnt/PCP pathway, Daam1 and ArhGEF19 (wGEF) perturb kidney tubulogenesis. Dishevelled, which activates both the canonical and non-canonical Wnt/PCP pathway, affect cilia formation in multiciliated cells. In this study, we investigated the role of the noncanoncial Wnt/PCP components Daam1 and ArhGEF19 (wGEF) in renal ciliogenesis utilizing polarized mammalian kidney epithelia cells (MDCKII and IMCD3) and *Xenopus laevis* embryonic kidney. We demonstrate that knockdown of Daam1 and ArhGEF19 in MDCKII and IMCD3 cells leads to loss of cilia, and Daam1’s effect on ciliogenesis is mediated by the formin-activity of Daam1. Moreover, Daam1 co-localizes with the ciliary transport protein Ift88 and is present in cilia. Interestingly, knocking down Daam1 in *Xenopus* kidney does not lead to loss of cilia. These data suggests a new role for Daam1 in the formation of primary cilia.

## Introduction

Primary cilia are microtubule-based cellular protrusions that allow a cell to sense its environment [1]. Many cell types in the body contain cilia, and improper cilia development results in a family of diseases called ciliopathies, including polycystic kidney disease, nephronophthisis, Joubert syndrome and Bardet–Biedel syndrome [2]. Although ciliopathies can manifest in a number of different ways, the vast majority of ciliopathies result in kidney abnormalities such as cystic kidneys, or nephronophthisis [3]. For example, one of the first identified ciliopathies is the result of a mutation in the gene *Ift88/Polaris*. Ift88 is a protein that is transported within ciliary vesicles to facilitate ciliary biogenesis [4,5]. Loss of Ift88 leads to cystic kidney disease in both mouse and human [6,7]. Additionally, loss of Ift88 in human mesenchymal stem cells leads to increased Wnt signaling and defects in planar polarity [8,9].

The canonical Wnt pathway has many connections to cilia development. In the zebrafish, loss of Lef1 or Tcf7, which are Wnt co-transcription factors, results in shorter and fewer cilia within the left/right organizer [10]. In mouse and *Xenopus laevis* a protein called Chibby1, which functions to shuttle β-catenin out of the nucleus, localizes to the cilia and is required for proper ciliogenesis and kidney development [11,12]. Additionally, mutations in genes that regulate cilia formation typically results in increased sensitivity to Wnt ligands, although the mechanism for this phenomenon is heavily debated [13]. Moreover, Dishevelled, a component of both the canonical and non-canonical Wnt pathway, is required for actin assembly and positioning of the basal bodies on the surface of the cell during ciliogenesis in *X*. *laevis* multiciliated skin cells [14].

Actin filaments are important for proper ciliogenesis. In motile multiciliated cells, the cilia are connected by an actin network [15]. Although this actin network is not clearly visible around primary cilia, and actin stains such as phalloidin do not detect F-actin within the cilia, application of drugs that broadly inhibit or stabilize actin filaments typically results in longer primary cilia [16–18]. Formin proteins act to polymerize actin filaments. However, treatment with the formin inhibitor smiFH2 has been found to decrease the number and length of primary cilia [16]. This suggests a complex story in which different sub-populations of actin can have either a positive or negative affect on ciliogenesis.

Daam1 (Dishevelled Associated Activator of Morphogenesis 1) is a Diaphanous-related Formin homology (DRF) protein that functions through the non-canonical Wnt/PCP pathway. It is regulated in part by Wnt ligands through Frizzled receptors and Dishevelled [19]. Daam1 is associated with a number of cellular functions, such as directed cell migration, planar cell polarity (PCP) and endocytosis [20–22]. Developmentally, Daam1 is necessary for gastrulation and normal kidney development in *X*. *laevis* [23–25]. Additionally, it is present within the the actin-rich base of multiciliated epidermal cells of the *X*. *laevis* skin where it regulates the actin network that stabilizes cilia. Loss of Daam1 in motile cilia results in their loss of polarity [26]. Additionally, proteomic analysis of primary cilia isolated from mammalian IMCD3 cells identified Daam1 as a potential ciliary component [27].

The Diaphanous family of formin proteins is regulated by the Rho family of GTPases [28]. Daam1 is known to bind to RhoA, a protein that regulates cytoskeletal dynamics. However unlike other Diaphanous family formin proteins, Daam1 is thought to be more strongly regulated by Dishevelled than Rho [19]. Wnt signaling through Dishevelled can activate RhoA, a process that is regulated by the Daam1/ArhGEF19 (wGEF) complex [29]. In mice, *arhgef19* is expressed mainly in the intestine, liver, heart and kidney [30], and loss of either ArhGEF19 or Daam1 in *X*. *laevis* leads to kidney malformations, with the development of edema when Daam1 is lost in both kidneys [24,25].

In this study, we find that loss of the PCP component Daam1 negatively regulates ciliogenesis in MDCKII and IMCD3 cells. Daam1 knockdown within these mammalian kidney epithelial cells result in the formation of fewer cilia. Furthermore, knockdown of Daam1 within *Xenopus* embryonic kidneys does not appear to cause loss of cilia. However, we cannot exclude the possibility that their structure, organization and/or function is affected. Our data indicate that ciliogenesis in MDCKII cells depends on the formin activity of Daam1. Additionally, the Daam1 partner, ArhGEF19, is also required for proper cilia formation in MDCKII cells. Finally, we found that in MDCKII and IMCD3 cells Daam1 localizes to vesicles that carry ciliary components, further supporting its role in ciliogenesis.

## Results

### Loss of Daam1 results in a failure of kidney epithelial cells to ciliate

**Fig 1:**
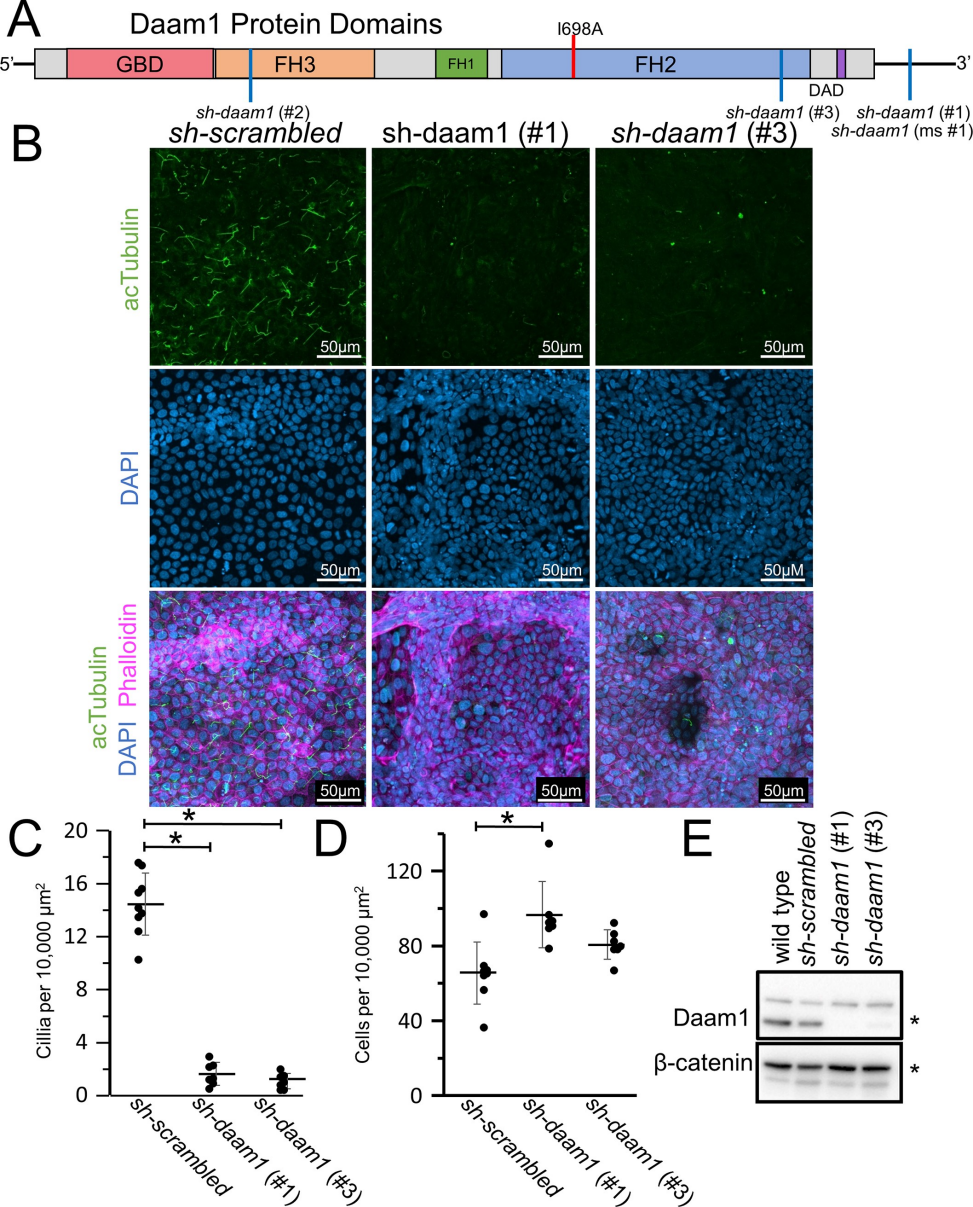
Daam1 knockdown results in a loss of primary cilia in MDCKII cells. **A)** Diagram illustrating the domains within the Daam1 protein. The GBD (DiaphanousGTPase-binding Domain), FH3 (Diaphanous FH3 Domain), FH1 (Formin Homology 1), FH2 (Formin Homology 2) and DAD (Diaphanous Auto-regulatory Domain) are indicated. The location of the I698A mutation within the formin homology 2 domain (FH2) is marked with a red line, and positions corresponding to the shRNAs are marked with a blue line. MDCKII canine kidney epithelial cells were infected with either *sh-daam1* or a control construct and grown upon transwell filters. **B)** Cells were stained with acetylated α-Tubulin antibody (acTubulin) to visualize primary cilia (green), DAPI to label nuclei (blue), and phalloidin to label F-actin (magenta). Confocal imaging was used to analyze the effect of Daam1 depletion on primary ciliogenesis. Scale bars equal to 50 μm. **C)** Cilia were quantified. Error bars are shown as ± SD and black dots indicate value of each image quatified. **D)** Cell numbers were quantified as described in S5 Fig. Error bars are shown as ± SD and black dots indicate value of each image quatified. **E)** Western blot of *sh-daam1* MDCKII cell lysates showing depletion of Daam1 protein levels. β-catenin was used as a loading control. * indicates p < 0.05 as compared to *sh-scrambled* using two tailed t test.

**Fig 2:**
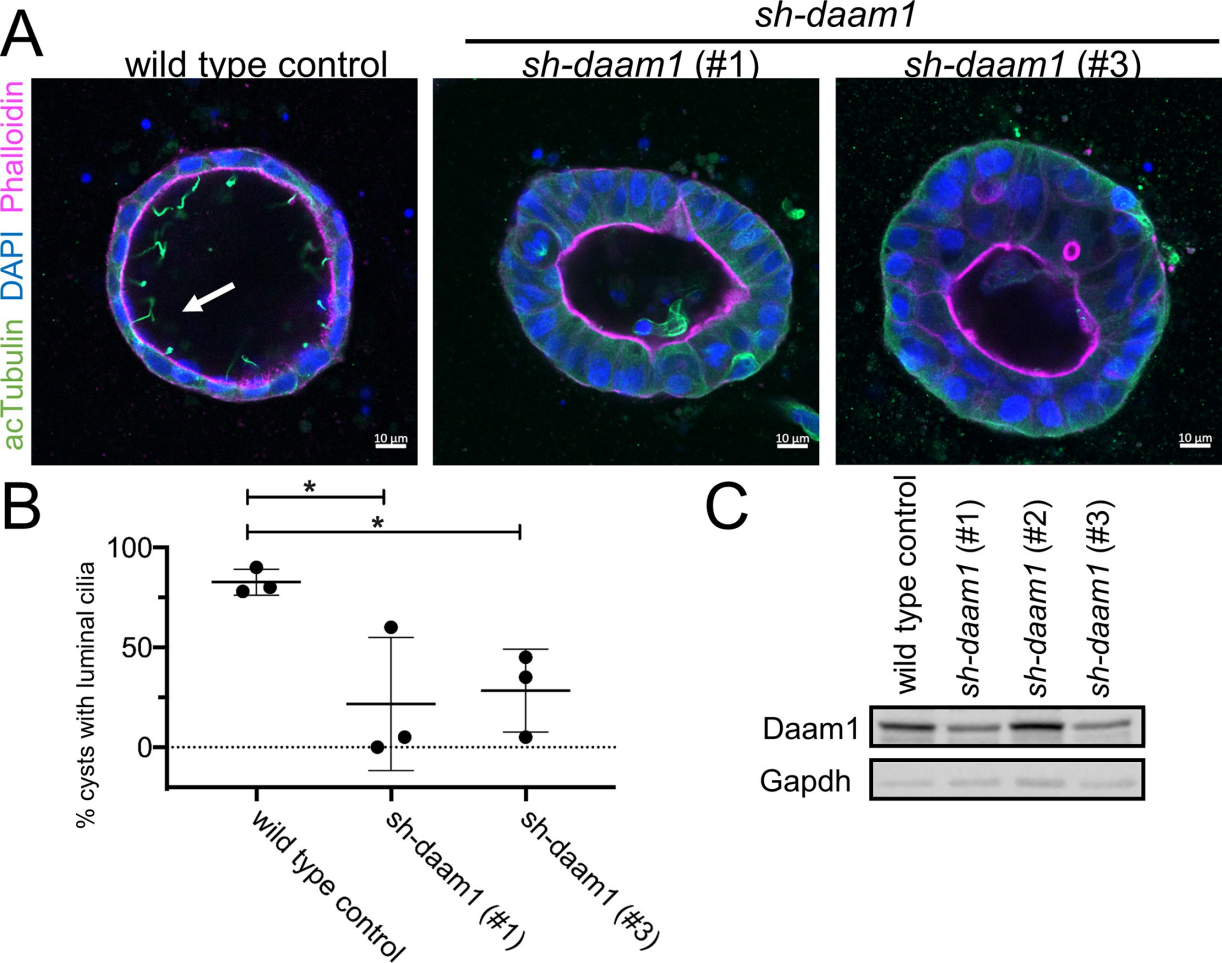
*sh-daam1* depleted MDCKII cells show reduced luminal ciliogenesis in 3D cultures. Control and *sh-daam1*-depleted cells were cultured in collagen I matrix to form cysts. After 12–14 days in culture, cysts were fixed and stained with an antibody against acetylated α-Tubulin (acTubulin) to visualize primary cilia (green), phalloidin for F-actin (magenta) and DAPI for nuclei (blue). Using confocal imaging we analyzed the effect of Daam1 depletion on ciliogenesis. **A)** Representative merged confocal images showing reduction of luminal cilia upon Daam1 knockdown. White arrow points to luminal cilia in control cysts. Scale bars equal to 10 μm. **B)** The graph shows quantification of cysts with luminal cilia for each condition. Twenty randomly chosen cyst per sample were analyzed in three independent experiments. Error bars are shown as ± SD; Significance was calculated using unpaired, two-tailed t-test; ns indicates p > 0.05, * indicates p < 0.05, **p < 0.01 **C)** Western blot showing Daam1 protein levels in wild-type (control) cells and in cells expressing different shRNAs targeting Daam1. GAPDH was used as loading control.

To determine whether Daam1 is required for primary ciliogenesis, *daam1* was knocked down in polarized MDCKII mammalian kidney cells. MDCKII cells form primary cilia when plated on a transwell membrane and are allowed to grow until confluency. Loss of Daam1 leads to cilia reduction in MDCKII cells (Fig 1). To ensure that this phenotype is due to loss of Daam1 we used two different shRNAs: one targeting the 3’UTR (#1) and one targeting the coding sequence of *daam1* (#3). Both shRNAs reduce Daam1 protein expression as visualized by Western blot (Fig 1E). A third shRNA, *sh-daam1*(#2), failed to reduce Daam1 expression (Fig 2C). Additionally, similar experiments were carried out in IMCD3 cells using a *sh-daam1*(#1) construct adapted for mice called *sh-daam1(ms#1)*. These experiments also indicate that knockdown of *daam1* disrupts primary ciliogenesis (S1A and S1B Fig).

Not only do *sh-daam1* cells have fewer numbers of cilia, but the cells grow to a higher density and exhibit a tendency to grow on top of each other, rather than forming a cell monolayer. When wild type cells pile on top of each other, they tend not to ciliate. Therefore, in our data analysis, we avoided scoring areas where cells failed to form a single monolayer.

MDCKII cells are used extensively to study renal tubulogenesis because they are capable of forming tubule-like structures when cultured in three-dimensional (3D) gels. These structures appear as single-layered, polarized, and hollow spherical cysts that closely reassemble the architecture of nephric tubules. Epithelial cells within cysts display apico-basal polarity and form luminal cilia similar to that of a nephron [31]. Ciliogenesis is a complex process that may involve the interaction of cells with the extracellular matrix (ECM) [32]. Thus, to further investigate the role of Daam1 in ciliogenesis, we cultured control and *sh-daam1*-depleted cells in collagen I matrix. While Daam1 knockdown cells were capable of forming 3D cysts, primary ciliogenesis was perturbed (Fig 2A and 2B). We observed a significant reduction of luminal cilia in Daam1-depleted cysts compared to controls (Fig 2A and 2B). Moreover, Daam1-depleted cysts displayed increased ciliogenesis on the basal–ECM facing side (S2A and S2B Fig). These results suggest that Daam1 is involved in regulating ciliogenesis and may be important for establishment and/or maintenance of apico-basal polarity. Additionally, we found that Daam1-depleted cells were more likely to form single lumens and present with cells within luminal space in comparison with controls (S2A and S2B Fig) and have some apical basal polarity defects as detected by phalloidin staining.

### Daam1 formin activity is required for ciliogenesis

**Fig 3:**
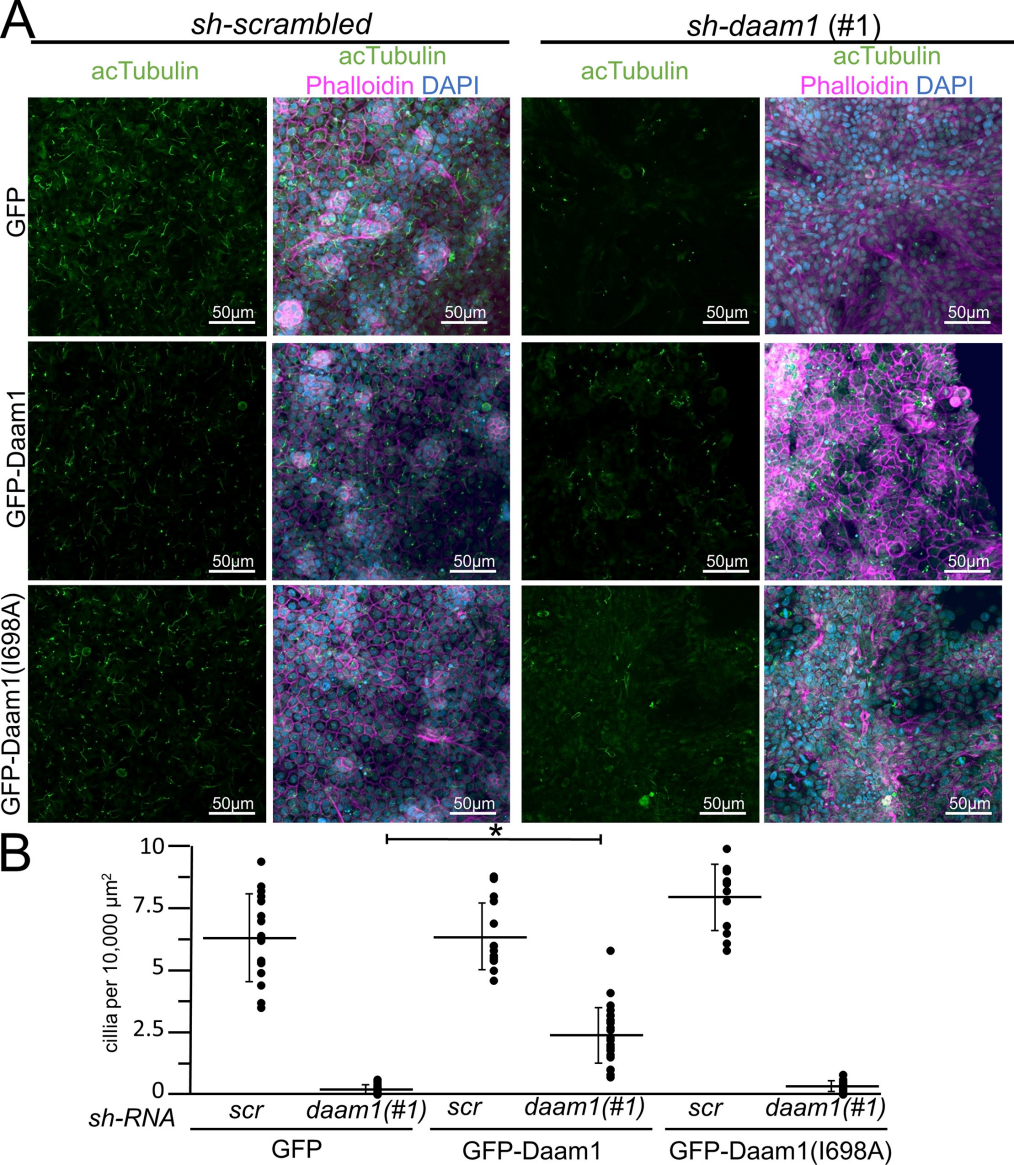
The *sh-daam1* cilia phenotype can be rescued with wild-type Daam1 but not Daam1(I698A). Stable MDCKII cells infected with either sh-daam1 or a control sh-scrambled construct were transiently transfected with constructs that express either GFP, GFP-Daam1, or GFP-Daam1(I698A) constructs. Cells were grown on transwell filters and stained with acetylated α-Tubulin antibody (acTubulin) to label primary cilia (green), DAPI to label nuclei (blue), and phalloidin to label F-actin (Magemta). Confocal imaging was used to analyze the effects upon primary ciliogenesis. **A)** Representitive images of ciliated cells stained with acetylated tubulin (green), dapi (blue) and phalloidin (magenta). Scale bars equal to 50 μm. **B)** Quantification of the amount of ciliation. Error bars are shown as ± SD. Black dots indicate individual values for each image quantified. * indicates p < 0.05 as compared to *sh-scrambled* using two tailed t test.

Given prior studies indicating that Daam1 functions on the actin cytoskeleton that stabilizes multicilia in *X*. *laevis* skin, we hypothesized that the formin activity of Daam1 is required for ciliogenesis. Rescue experiments were carried out to investigate *sh-daam1* cilia phenotype using constructs that express either GFP-Daam1 or a GFP-Daam1(I698A) mutant, which disrupts the actin binding activity of the FH2 domain [33,34]. Due to an inability to generate stable Daam1 expressing lines, we utilized transient transfections to express wild type Daam1 or Daam1(I698A). Approximately 30% of the cells had strong GFP expression. Wild type Daam1 partially rescued the reduced cilia phenotype (Fig 3). In contrast, the Daam1(I698A) mutant failed to rescue the cilia phenotype.

**Fig 4:**
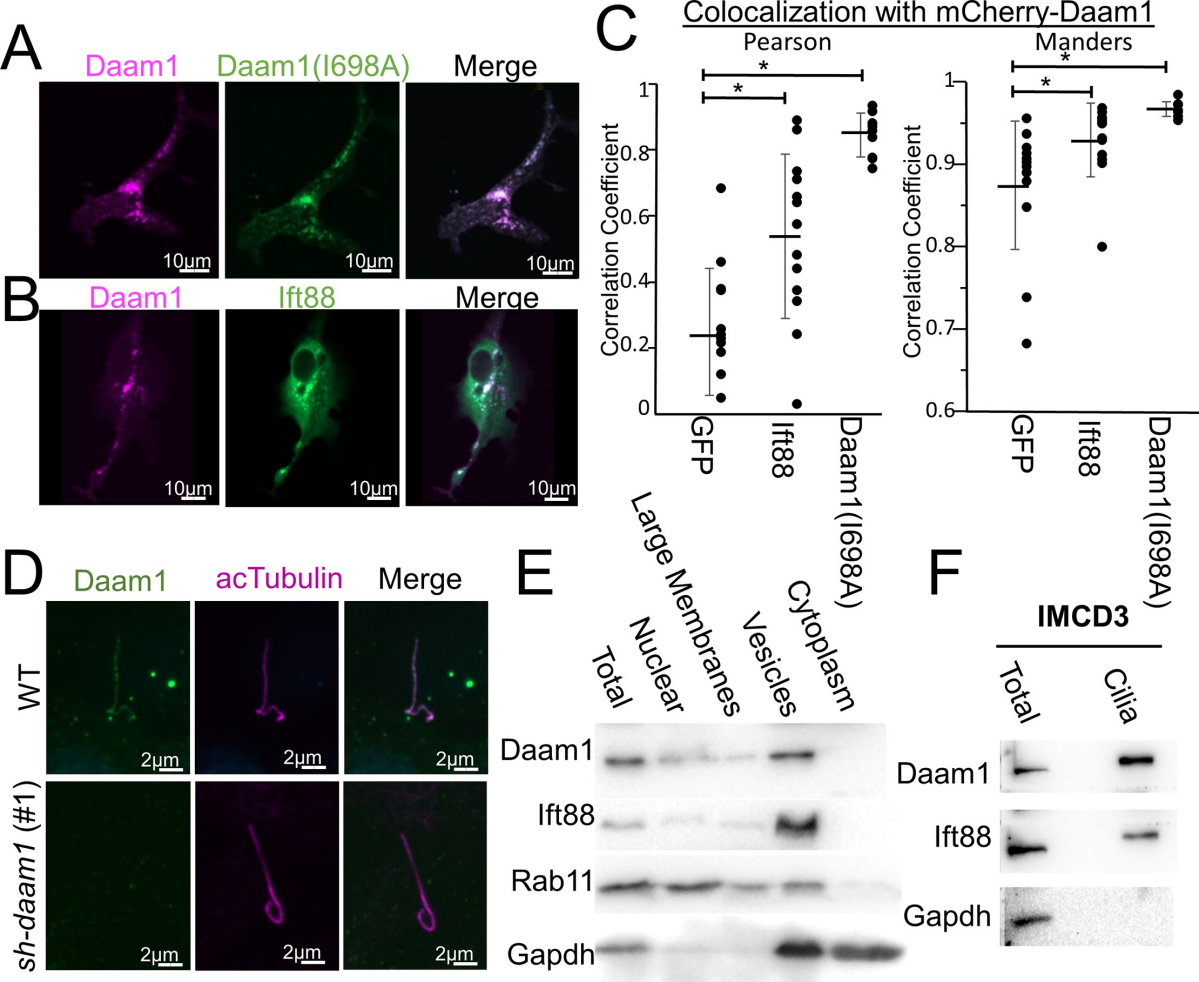
Daam1 localizes to ciliary vesicles and cilia. **A)** MDCKII cells were co-transfected with constructs that express mCherry-Daam1 and GFP-Daam1(I698A) then imaged via confocal to analyze the localization in live cells. **B)** MDCKII cells were co-transfected with constructs that express mCherry-Daam1 and Ift88-GFP than imaged via confocal in live cells to determine the subcellular localization. Scale bars equal to 10 μm. **C)** MCDKII Cells were transfected with mCherry-Daam1 along with GFP, GFP-Ift88, or GFP-Daam1(I698A), then colocalization was measured using both the Pearson and Manders equations. Error bars are shown as ± SD and black dots indicate colocalization value for each cell measured. **D)** Wildtype and *sh-daam1* (#1) cells were cells were ciliated, fixed, then stained for Daam1 (Proteintech) and acTubulin (Sigma). **E)** MDCKII cells were subconfluently grown then fractionated for subcellular components. “Total” indicates whole cells lysated isolated prior to fractionation. Lysates were ran on Western blot and probed for Daam1, secretory components (Rab11), ciliary vesicles (Ift88), and cytoplasmic + vescular components (GAPDH). **F)** Cilia was isolated from IMCD3 cells and lysates were ran on a Western blot. “Total” indicates whole cell lysated isolated prior to deciliation. The blot was probed for the Daam1, a ciliary marker Ift88, and a cellular marker GAPDH.

Because the Daam1 formin mutant failed to rescue loss of cilia and some Daam1 truncations such as Daam1(524–1078) fail to localize properly [21], GFP-Daam1(I698A) localization was visualized to determine whether it inability to rescue the *sh-daam1* phenotype was due to mislocalization of the protein. mCherry versions of these constructs were made, then co-transfected. The wild type and I698A versions of Daam1 were found to colocalize with each other (Fig 4A and 4C).

### Daam1 localizes to ciliary vesicles

To further examine the role of Daam1 in ciliogenesis, the subcellular localization of Daam1 was observed in live cells transfected with GFP-Daam1. In both ciliated and unciliated cells Daam1 predominantly localizes to highly motile puncta (Fig 4A and 4B, S1D Fig, S3A and S3B Fig). To assess whether Daam1 is localized at the cilia, its localization was visualized concurrently with that of ciliary markers. Live cells were utilized to assess Daam1 localization. However, because MDCKII cells are polarized on an opaque membrane and a high level of autofluorescence appears after 5 days of confluency, MDCKII cells are not ideal for live imaging of cilia. IMCD3 cells start to ciliate within 24 hours of confluency and are normally fully ciliated within 2 days, therefore, transiently transfected IMCD3 were ciliated on glass coverslips to directly visualize cilia in live cells. A GFP-Chibby1 construct was used to label the transition zone of the cilia and an α-Tubulin-GFP construct was used to label the axoneme of the cilia (S3A and S3B Fig) [35]. Using these constructs, co-localization of Daam1 with the ciliary transition zone or axoneme of cilia was not apparent.

Because vesicles are involved in cilium biogenesis, and the puncta seen in Daam1 overexpression experiments are possibly vesicles, localization studies were carried out to determine whether Daam1 puncta are vesicles that carry ciliary components. To test this hypothesis we cotransfected mCherry-Daam1 with Ift88-GFP constructs in both IMCD3 and MDCKII cells. Ift88 is a marker of ciliary vesicles that are being transported to the cilia [5]. As Ift88 vesicles are hard to detect outside of the cilia in cilated cells, we transfected subconfluent unciliated cells to examine the colocalization between Daam1 and Ift88. We found that vesicles labeled with Ift88-GFP colocalized with mCherry-Daam1 (Fig 4B and 4C and S1C and S1D Fig). Many Ift88-GFP transfected cells expressing did not have identifiable vesicles; therefore, these cells were not used for analysis as ciliary vesicles could either not be seen or did not exist at this point in cliliary developement. Both the Ift88 and Daam1 labeled vesicles present in MDCKII cells are much smaller and more numerous than the vesicles in IMCD3 cells (Fig 4B and S1C and S1D Fig). Additionally airyscan superresolution imaging of the vesicles in IMCD3 cells suggests that Daam1 is localized to the vesicle membrane (S3F Fig). However as Daam1 is not predicted to have a transmembrane domain or a signal peptide we predict that Daam1 is associated with the outside of the vesicle.

Since overexpression can result in mislocalization of proteins, we carried out subcellular fractionation studies in MDCKII cells to verify that endogenous Daam1 is associated with vesicles. We used Ift88 as a verified ciliary vesicle marker [5]. Other markers were used to help verify the content our fractions Rab11 is a component of the secretory pathway and is associated with a large number of membrane components [36]. GAPDH is used as a marker of cytoplasm and COPI vesicles [37]. By subcellular fractionation, Daam1 is found in nuclear, large membrane, and small vesicle fractions, and it is absent from the cytoplasmic fraction (Fig 4E). The relative amount of Daam1 found in the nuclear and large membrane fractions varied from trial to trial but was always detectable.

### Daam1 is found in the cilia

As further validation of Daam1 localization we immuniostained both MDCKII cells and IMCD3 cells for Daam1. We obtained three different antibodies that were generated against Daam1 (Proteintech, Abnova, Biorad). Two of the antibodies were independently generated mouse monoclonal antibodies that target the first ~110 amino acids of Daam1, while the other one was a rabbit polyclonal that targeted the last 353 amino acids of Daam1. By immunostaining, all three antibodies detected Daam1 in the cilia. Daam1 and Ift88 largely do not colocalize within the cilia; however, there are a puncta in which colocalization can be detected (Fig 4D and S3C and S3D Fig). Formalin and Dents fixation did not produce a convincing signal from any of the antibodies tested however glyoxal fixation produced a convincing signal from the cilia for all three antibodies. All three antibodies are able to detect Daam1 overexpression. Control samples to which primary antibody was not added, failed to stain the cilia. Additionally loss of Daam1 results in a reduction of Daam1 signal in the cilia, though it was rarely completely missing (Fig 4D).

As the antibody staining and overexpression of Daam1 showed a different expression pattern we wished to have another means of validating the antibody signal. Threfore, we purified out cilia using the calcium shock method and ran the protein lysates on a Western blot. An Ift88 antibody was used as a ciliary marker and a GAPDH antibody was used to detect possible contaminating cytoplasm and vesicles that could co-purify with the cilia. A strong Daam1 signal was detected in the purified cilia (Fig 4F). There was also little to no detectable GAPDH signal in the ciliary fraction.

### ArhGEF19 is required for ciliogenesis

**Fig 5:**
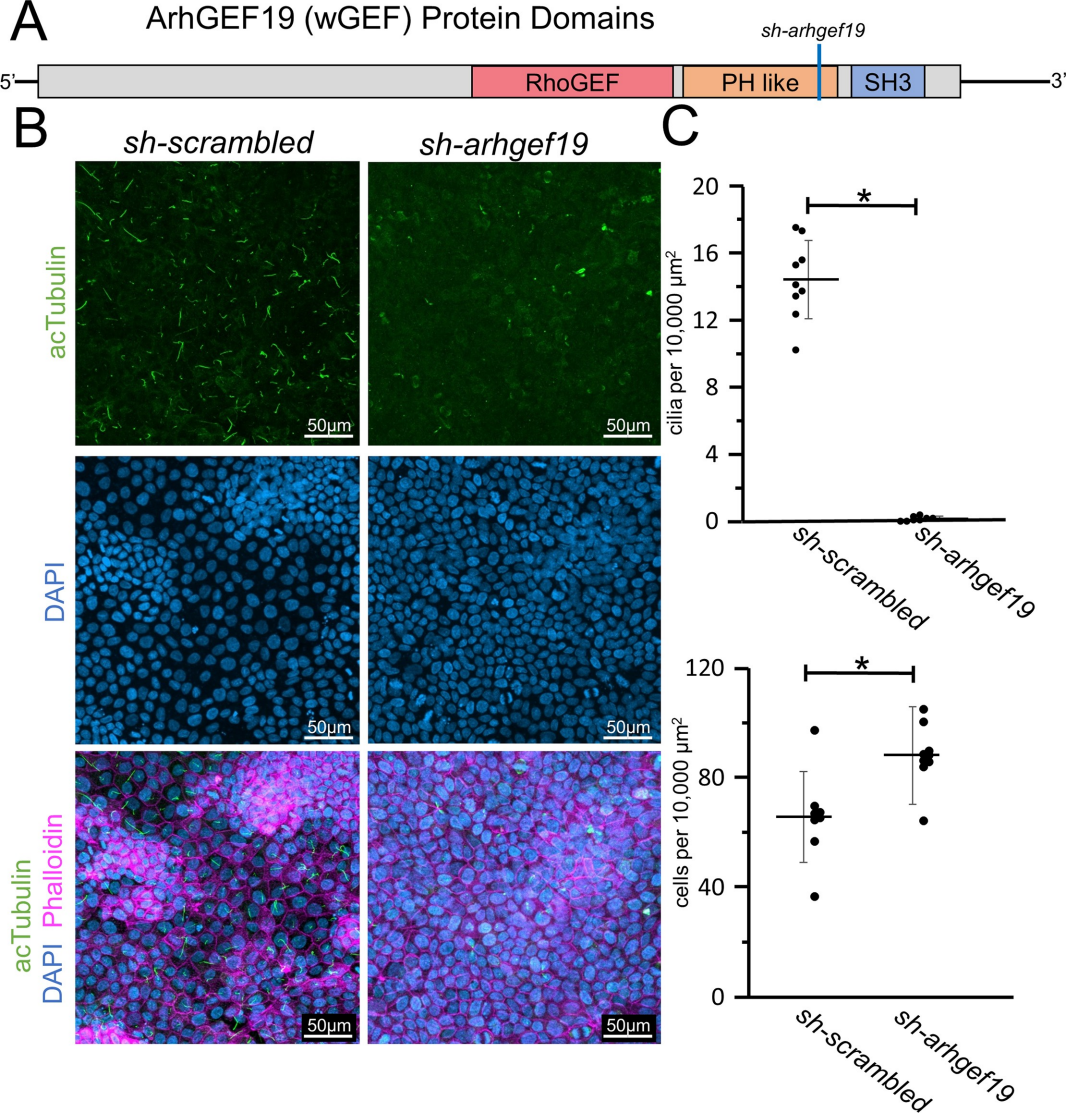
Arhgef19 knockdown results in loss of primary cilia in MDCKII cells. **A)** Diagram of the domains within the Arhgef19 (WGEF) protein. The corresponding position of the shRNA is marked with a blue line. **B)** MDCKII cells were infected with either a control construct or a construct that targets Arhgef19 and then polarized on transwell filters. Cells were stained with acetylated α-Tubulin antibody (acTubulin) to visualize primary cilia (green), DAPI to label nuclei (blue), and phalloidin to label F-actin (magenta). Confocal imaging was used to analyze the effects Arhgef19 depletion upon primary ciliogenesis. Scale bars equal to 50 μm. **C)** The number of cilia was counted and cell numbers were quantified as described in S5 Fig. * indicates p < 0.05 as compared to *sh-scrambled*. Error bars are shown as ± SD and black dots indicate value of each image quatified.

As loss of Daam1 results in loss of cilia, we assessed whether ArhGEF19, a RhoGEF that associates with Daam1 and acts within the non-canonical Wnt/PCP pathway, is required for ciliation. If RhoA activation is required for ciliogenesis, loss of ArhGEF19 should mimic the phenotypes shown in *sh-daam1* cells. Loss of *arhgef19* results in almost complete loss of cilia (Fig 5). Similar phenotypes were observed in *sh-arhgef19* cells and *sh-daam1* cells, with *sh-arhgef19* cells growing to higher density and exhibiting cilia loss. However, one distinction noted was that in contrast to *sh-daam1* cells, the *sh-arhgef19* cells do not grow on top of each other, indicating that distinct mechanisms may also exist. The loss of cilia upon *arhgef19* depletion indicates that GTP-RhoA as part of the PCP pathway is necessary for ciliogenesis.

### Loss of Daam1 in *X*. *laevis* kidney does not cause loss of primary cilia

**Fig 6:**
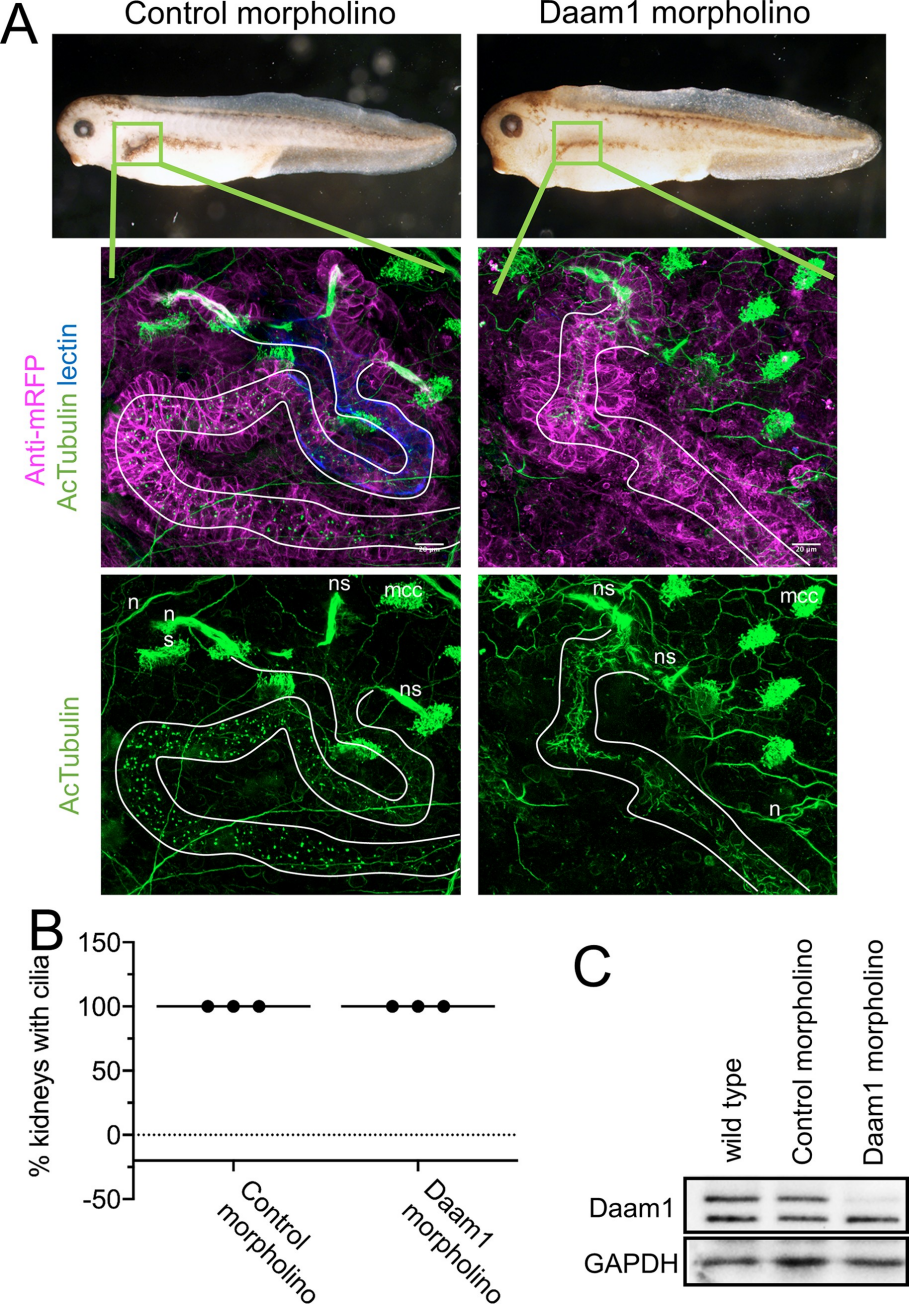
Daam1-depletion does not cause absence of cilia within *Xenopus* embryonic kidneys. Because knockdown of Daam1 in *Xenopus* kidney leads to kidney defects, we analyzed the effect of Daam1 knockdown on renal ciliogenesis. We injected Daam1 or Standard (Control) morpholino in combination with a membrane-tagged red fluorescent protein (mRFP) mRNA as a lineage tracer, into a *Xenopus* blastomere fated to the nephric anlagen. Embryos were fixed at stage 39–40 and stained with an antibody against acetylated α-Tubulin (acTubulin) to label cilia (green), anti-mRFP lineage tracer (magenta) and lectin to label the proximal tubule (blue). **A)** Stereoscope brightfield imaging shows the gross morphology of Control and Daam1-morpholino injected embryos. Confocal fluorescent imaging of boxed regions (green) shows magnified views of corresponding kidneys. Kidney tubules displaying primary cilia are outlined in white. Neurons (n), multiciliated epidermal cells (mcc) and multiciliated cells within nephrostomes (ns) are immunostained with acetylated α-Tubulin (acTubulin) antibody. Scale bars equal to 20 μm. **B)** The graph represents the percentage of Control (n = 32 embryos) and Daam1-depleted (n = 34 embryos) kidneys with primary cilia. **C)** Western blot showing Daam1 protein expression levels in uninjected wild-type, control and Daam1 morphant embryos.

Daam1 activity has been shown to be required for proper nephron development in both *Xenopus* and zebrafish embryos [24,25]. Given that changes in nephron morphology and ciliogenesis are closely linked, *Xenopus* embryos were used to assess the role of Daam1 in formation of renal primary cilia. To avoid gastrulation defects associated with manipulating Daam1 expression in the whole embryo, a well-established kidney-targeted-morpholino approach was employed to knock down Daam1 activity in *Xenopus* nephric progenitors [24,25,38]. This approach consists of co-injecting Daam1 morpholino, or control morpholino, with mRNA encoding membrane-bound fluorescent protein (tracer to confirm correct delivery). Injections are made into selected blastomeres at the 8-cell stage that are fated to give rise to the kidney. As expected, Daam1 morpholino injected embryos show defects in nephron formation. Intriguingly, loss of primary renal cilia upon Daam1 knockdown was not observed (Fig 6, and S4 Fig), as it was observed in MDCKII and IMCD3 cells following Daam1 depletion. While pronephric depletion of Daam1 leads to reduced elaboration of proximal and distal nephric tubules in stage 40 embryos, this phenotype is much less developed during early stages of nephron formation. To exclude the possibility that an earlier loss of renal tubules masks the loss of cilia phenotype, we also examined cilia in stage 30 embryos upon Daam1 knockdown. However, even in early stage embryos, there was no apparent loss of primary cilia upon Daam1 depletion (S4 Fig). These data suggest that there are compensatory mechanisms that function *in vivo* to ensure proper ciliogenesis upon Daam1 knockdown.

## Discussion

Prior work demonstrated that within multicilliated cells of the *Xenopus* skin Daam1, a component of the planar cell polarity pathway, is important for the proper development of motile cilia [26,39]. Additionally, the cilia have been found to be important for cell polarity and cell divisions [9,40]. In multiciliated cells Daam1 forms the actin network for proper anchoring of the basal bodies. However, a role for Daam1 in formation of primary sensory cilia has not previously been identified. In this study, we analyzed the role of Daam1 in renal primary ciliogenesis.

To be consistent, MDCKII cells were used whenever possible. However, because certain experiments are difficult to perform in MDCKII cells, IMCD3 cells were utilized in some cases. Given that depletion of Daam1 in IMCD3 cells also disrupts ciliogenesis and Daam1 colocalizes with Ift88 in IMCD3 cells, we predict that Daam1 is functioning through a similar mechanism in both MDCKII and IMCD3 cell lines (S1 Fig). Two experiments in this study required the use of IMCD3 cells. The first is live imaging of cilia, given that MDCKII cells are ciliated on an opaque membrane this makes visualization difficult (S3 Fig). The other experiment is the isolation of cilia; curent protocols are developed for isolation of cilia in IMCD3 cells [41,42]. Since MDCKII cells do not ciliate well on plates, these protocals are not easily transferable to MDCKII cells (Fig 4). All other experiments were performed in MDCKII or both MDCKII and IMCD3 cell lines.

Within the three models examined, the degree to which Daam1 influences ciliogenesis is variable. 2D *daam1*-depleted MDCK cultures show absence of cilia, while 3D cultures have a weaker phenotype and display reductions in luminal ciliogenesis. *X*. *laevis* Daam1-depleted embryonic kidneys are morphologically abnormal but still have renal cilia. An almost identical scnerio is seen with studies looking at Exoc5/Sec10 protein. In 2D cultures MDCKII cells knockout of Exoc5 results in a reduction of cilia [43]. While in 3D cysts there is a falure to form a monolayered cyst, cilia can still be formed [44]. Mouse and Zebrafish models have kidney abnormalities but still have cilia [44,45]. Exoc5 is not unique in this aspect with similar phenotypes seen with Dnmbp depletion of Dnmbp in MDCKII cells prevents ciliogenesis, while in zebrafish and *X*. *laevis* morphants for Dnmbp do not exhibit loss of cilia [31,46]. Even for the more established ciliary genes it is rare to see a complete loss of cilia in animal models. Also within the same cell line, experiments studying the affects of Rac1 on cell polarity show different phenotypes within 2D and 3D cultures [47]. The more common phenotype is either a reduction in number or a shortening of cilia, similar to the phenotypes we see with Daam1. Given that 2D MDCKII cells ciliate upon confluence, IMCD3 cells require both confluence and serum starvation, and *X*. *laevis* kidney progenitor cells form cilia prior to epithelization, distinct factors within each model likely play roles in their ciliation. This divergence in phenotype suggests that other factors may compensate for loss Daam1 and are likely to be influenced by extrinsic factors. For example, other formin proteins may facilitate actin polymerization to stabilize cilia or transport vesicles in the absence of Daam1 *in vivo*. Alternatively, elements of the extracellular environment, such as extracellular matrix components, may compensate for Daam1 loss in embryos. Alternatively, given that Daam1 influences the positioning of motile cilia in *X*. *laevis* epidermal cells [26], it is possible that similar defects occur in renal primary cilia that are not detectable by immunostaining.

It has previously been reported that Daam1 localizes to endocytic vesicles, actin filaments and the cell membrane [20,21]. Based upon our finding that Daam1 localizes to vesicles that carry ciliary components. There are a couple of potential mechanisms to explain how Daam1 plays a role in ciliogenesis. For example, Daam1 may be involved in vesicle trafficking to the base of the cilium or the docking of vesicles carrying ciliary components to the basal body. In *sh-daam1* 3D cysts, the few cilia that are formed sometimes contain ciliary bulges. A similar phenotype is seen with a number of ciliary genes in mammals such as Chibby1 and the Bardet–Biedl proteins BBS2 and BBS4 [48,49]. Although, the primary function of the Bardet–Biedl proteins is vesicular trafficking of proteins to the cilium, some of the BBsome subunits are thought to associate with IFT particles to regulate vesicular trafficking within the cilia [50]. In *Chlamydomnonas* loss of the IFT-A transport complex subunits similarly results in ciliary bulges [51]. Though Daam1 is found in Ift88 labeled vesicles in unciliated cells we do not have any evidence that they directly interact.

There are several explanations as to why we were not able to detect Daam1 in cytoplasmic vesicles by antibodies in unciliated cells that were observed by overexpression and cell fractionation. As our antibodies only poorly detect Daam1 by immunofluorescence, it is possible that these vesicles do not carry enough Daam1 to be detected by these antibodies. This is also counfounded by the fact that glyoxal fixation results in bright autoflorescent spots in the cytoplasm which may be hiding the real Daam1 signal in the cytoplasm. We did test these antibodies on cells overexpressing Daam1, and then the vesicles were visible (data not shown).

Although we were able to detect Daam1 in purified cilia fractions and by immunostaining using three different antibodies, we were not able to detect Daam1 in or near the cilia by overexpression. Other groups have found Daam1 in cilia by large scale analysis of ciliary proteins, providing further evidence that Daam1 is present within the cilium [27,52]. Even though multiple formin proteins have been found in the cilia, their functions are not clear given that F-actin is not detectable within the axoneme of the cilia, suggesting that these formin proteins may not function within the axoneme itself [16,52]. In MDCKII cells DSH2 and DSH3 are present at the cilia base near the basal body [53]. It is possible that Daam1 is present but inactive, and only actived upon leaving the cilia. The purpose of actin around the cilia is to function in endocytosis of ciliary vesicles, exocytosis of preciliary vesicles and anchoring of the basal bodies in multiciliated cells [54]. It is possible that Daam1 regulates some or all of these processes by regulating the actin cytoskeleton around the cilia. It is also possible that Daam1 is directly involved in vesicular trafficking to, within and from cilia.

Because cells lacking Daam1 have multiple phenotypes, identification of just one mechanism by which Daam1 influences ciliogeneisis is not feasible. It is possible that loss of cilia is a secondary to other phenotype caused by loss of Daam1, such as polarity defects observed in the 3D cysts. As *sh-daam1* cells grow to a higher density they may also have problems with contact inhibition and never completely leave the cell cycle an thus may never differentiate to form primary cilia.

The requirement of both ArhGEF19 and Daam1’s formin activity for ciliogenesis indicates a dual role for Daam1 in ciliogenesis through both actin polymerization and RhoA activation [33,34]. While future studies will be required, it is possible that ArhGEF19 functions through a Daam1-independent mechanism to promote ciliogenesis. Given that no other proteins that affect ArhGEF19 activity have been identified and the effector proteins of Wnt-activated RhoA signaling are currently unknown, it is difficult to disprove this hypothesis. Our data indicate a similar cilia phenotype upon *daam1* and *arhgef19* depletion. However, *sh-daam1* cells tend to grow on top of each other, while *sh-arhgef19* cells do not, suggesting unique deficits in cell migration and epithelization in the *sh-daam1* cells. Thus, subsequent study will be needed to elucidate the role of ArhGEF19 in ciliogenesis.

Overall, our work indicates that the planar cell polarity effector, Daam1, promotes the formation of primary cilia in MDCKII and IMCD3 kidney epithelial cells. The formin activity of Daam1 and the Daam1-associated GEF, ArhGEF19, are required for proper cilia formation. Given prior work in *X*. *laevis* skin indicating Daam1 affects the polarized orientation of motile cilia [26], it is possible that organization of the primary cilia within the kidney is also affected, but due to complexity of kidney morphology in 3D, we unable to detect such changes. However, our data showing localization of Daam1 to vesicles that carry ciliary components indicates a novel mechanism by which Daam1 may play a role in ciliogenesis.

## Materials and methods

### Cell culture

All cells were grown at 37°C with 5% CO2. Cells grown for experiments were passaged prior to confluency, as confluent cells required excessive trypsinizing resulting in problems with ciliation. MDCKII cells were grown in DMEM + 10% FCS + Antibiotic/Antimycotic solution (Sigma A5955) [55] and IMCD3 cells were grown in DMEM/F12 + 10% FCS + Antibiotic/Antimycotic solution [42]. MDCKII and IMCD3 cell were purchased from ATCC.

### Transfections and stable cell line generation

pLKO.1 lentiviral construct along with packaging vectors (ΔVPR and VSVG) or (psPAX2 and pMD2.G) were transfected using Fugene-6 (Roche) or PEI (Polysciences 23966–2) into HEK293T cells grown on a 10 cm dish [56]. Supernatant containing transfection reagent was discarded and replaced with 5 ml of fresh media. Supernatant was collected and replaced with 5 ml fresh media over the course of 3 days resulting in 25 ml of virus. The virus was filtered through 0.2 μm filter to remove debris and frozen in 5 ml aliquots at -80°C.

Cells to be infected were split and allowed to grow to 50% confluency in a 10 cm plate then washed with PBS. Virus was thawed at 37°C and 5 μl 10 mg/ml polybrene (Sigma H9268) was added to the virus, then the entire 5 mls were added to cells. Cells were allowed to incubate with virus for 24 hours. Virus was then washed off and replaced with DMEM and the cells were allowed to grow for an additional 24 hours. DMEM + 10 μg/ml puromycin was then added to the cells and they were allowed to grow to confluency. Media was changed daily to remove cell debris.

Transient transfection was performed on uncoated glass coverslips or 6 well dishes using 5 μg of plasmid along with 30 μg polyethylenimine, linear mw 25,000 (Polysciences 23966–2). Cells were washed with DMEM or DMEM-F12 2–4 hours after transfection reagent was added [57].

### Ciliation

For MDCKII 2D cell culture, cells were diluted 1:10 from a nearly confluent plate onto plastic dishes and then allowed to grow overnight. 2.5x10^4^ cells were then plated onto transwell filters (Corning 3460) with DMEM on both sides of the filter. Cells were confluent the day after plating and were allowed to grow for an additional 4–5 days to ciliate. Media was changed every other day. For rescue experiments, cells were transfected in 6 well dishes and expression was verified prior to moving to transwell filters.

MDCKII cyst formation was modified from previous work [58,59]. Cells were diluted 1:10 from a nearly confluent plate onto plastic dishes and then allowed to grow overnight. 8-well cell chambers (Falcon 08-774-26) were precoated with 85 μl collagen solution (24 mM glutamine, 2.35 mg/ml NaHCO_3_, 20 mM HEPES pH 7.6), 1X MEM, 2 mg/ml type I collagen (Corning 354249)]. Cells were collected and strained through a 0.4 μm strainer to remove cell clumps. 2.6 x10^3^ cells were resuspended in 175 μL collagen solution and placed in the precoated cell chambers. 400 μL DMEM was add to each chamber after the collagen solidified. DMEM was changed once every other day for 12–14 days.

For IMCD3 cells, 2.5 x 10^4^ cells were plated onto glass coverslips and allowed to grow to confluency. Media was washed off and replaced with DMEM-F12 without serum for two days.

Cilia counts were performed by manually counting all cilia within an image. *sh-daam1* cells tend to pile on top of each causing automated methods of counting cells to miscount cells. For this reason, we split images up into a 4 x 4 grid and manually counted the same four sections in each image (S5 Fig).

### Staining and imaging

#### 2D MDCK and IMD3 cell cultures

For cilia counts cells were fixed in 4% PFA in PBS followed by 100 mM Glycine, and for Daam1 immunostaining cells were fixed using glyoxal fixative (4% glyoxal, 5%(v/v) ethanol, 130 mM acetic acid pH 4.5) [60]. Cells were blocked in 10% goat serum in PBS-T (PBS + 0.1% Triton-X). 1:1000 Phalloidin-Alexa568 (Invitrogen, A12380) was used for staining of F-actin, and 5 μg/ml 4’6’-diamidino-2-phenylindole were used to detect nuclei. Primary antibody 1:1000 α-Tub1a (Sigma -T6793) and 1:500 α-Daam1(Biorad-MCA3766Z, AbNova H00023002-M05, or Proteintech -14876-1-AP) in combination with secondary antibody anti-mouse IgG Alexa 647 (Invitrogen, A-21235), anti-mouse IgG Alexa 488 (Invitroge (Invitrogen, A-11001), anti rabbit IgG Alexa 555 (Invitrogen, A21428), and or anti rabbit IgG Alexa 488 (Invitrogen, A11008).

#### 3D MDCK cultures

Staining of MDCK 3D cell cultures was carried out as previously described [55]. Slides were incubated with primary 1:1000 mouse α-Tub1a (Sigma -T6793) antibody and detected with 1:200 anti-rabbit CY3 (Jackson Immunoreserach, cat#715-165-151) or 1:200 Alexa488 Mouse (1:200, Jackson Immunoresearch, cat#715-545-150) secondary antibodies. F-actin was labeled with Alexa Fluor 647 Phalloidin (1:200, Invitrogen, A22287) while nuclei were detected with 4’6’-diamidino-2-phenylindole (1:500, DAPI). Slides were mounted in Flouromount-G medium (Southern Biotech, cat# 0100–01) prior to imaging.

Following immunostaining and mounting, the 3D MDCK cysts were subjected to quantitative analyses. For each condition, cysts were analyzed in random and repeated positions across the slide. All samples were visualized using a Nikon A1 laser scanning confocal microscope and 20x objective. Because viewing of the entire cysts often was not feasible due to their different positioning in Z-planes within the extracellular matrix, we focused our analysis on the mid- sections from the cysts.

#### *Xenopus* embryos

Embryos were staged [61], fixed in MEMFA (0.1 M MOPS pH 7.4, 2 mM EGTA, 1 mM MgSO_4_, 4% formaldehyde) [38] and immunostained using established protocols [62]. Primary antibody 1:100 mouse α-Tub1a (Sigma -T6793), 1:250 rabbit RFP (MBL International -PM005) and 1:250 rabbit Lhx1 (gift from Masanori Taira, [63]) were used. Proximal tubules were stained using fluorescein-coupled *Erythrina cristagalli* lectin at 50 μg/ml (Vector Labs). Secondary antibodies anti-mouse IgG Alexa 647 (Invitrogen, A-21235), anti-mouse IgG Alexa 488 (Invitrogen, A-11001) and anti-rabbit IgG Alexa 555 (Invitrogen, A-21428) were used at 1:500 concentration. Embryos were dehydrated in methanol and cleared in a benzyl benzoate/benzyl alcohol (2:1) solution for imaging.

#### Imaging

Images were taken using an Olympus SZX16 fluorescent stereomicroscope and an upright Leica SP5, inverted Nikon A1, and an inverted Zeiss LSM800 laser scanning confocal equipped with airyscan detector and cell incubation chamber. Nikon-Elements, Zen blue, ImageJ (Fiji plugin), Adobe Photoshop and Microsoft PowerPoint were used for data analysis and image processing. For live imaging cell were grown on coverslips and imaged in Attofluor^™^ Cell Chamber (Thermo A7816). Colocalization analysis was done on manually traced cells using Zen software (Zeiss) as per manufacturers instructions. The software calculates the Pierson and Manders Cooeficient as published in [64].

### Western blot

#### Cell lysates

Cells were trypsinized from plates, collected and washed twice in PBS prior to being resuspended in 2X Laemmli (Biorad) plus 100 μm dithiothreitol. The resulting cell lysates were then boiled at 95°C for 30 minutes. Lysates were run on an 8% SDS-PAGE gel and the protein was then transblotted onto a 0.2 μm nitrocellulose membrane (GE Healthcare), followed by blocking for 3 hours in KPL block (SeraCare) at room temperature. Blots were incubated in 1:1000 rabbit anti-GAPDH (Santa Cruz sc-25778), 1:1000 rabbit anti-Daam1 (Proteintech 14876-1-AP) or 1:1000 rabbit anti-β-catenin [65] primary antibodies for 1–2 hours. Blots were then washed with TBST, incubated in goat anti-rabbit IgG horseradish peroxidase secondary antibody (1:5000, BioRad) for 2 hours at room temperature and then washed again with TBST prior to imaging on a BioRad ChemiDoc XRS+ imaging system using enhanced chemiluminescence (Pierce Supersignal West Pico). Preparation of MDCK cell extracts and Western blot analysis presented in Fig 2 were carried out following the protocol described by [55].

#### Embryo lysates

1-cell *Xenopus* embryos were injected with 20 ng in 10 nl of Daam1 or Standard morpholino in combination with 0.5 ng mRFP. Lysates were prepared from Stage 11 embryos and Western blots were performed following previously established protocol [24].

### Subcellular fractionation

Subcellular fractionation was performed by a modified protocol obtained from Abcam <https://www.abcam.com/ps/pdf/protocols/subcellular_fractionation.pdf>. Cells were grown on 10 cm plates and harvested by scraping. Cells were washed 2 times in PBS and pellets were resuspended in 500 μl of 0.1X cell fractination buffer. Cells were passed through a 25 gague needle 10 times and centrifuged at 800 g for 5 minutes. The pellet contains the crude nuclear fraction and the supernatant was saved for other fractions. Nuclear pellet was resuspended in 1X cell fractination buffer (250 mM Sucrose, 10 mM KCl, 1.5 mM MgCl_2,_ 1 mM EDTA, 1 mM EGTA, 1 mM DTT, 1 mM PMSF, 20 mM HEPES pH 7.4) and passed through 25 gague needle 10 more times, followed by centrifugation at 800 g for 5 minutes. Supernatant was discarded and pellet was washed one more time in 1X cell fractionation buffer, discarding supernatant. The nuclear pellet was resuspended in 200 μl laemmli buffer. To collect large membrane fraction, the supernatant from above was centrifuged at 7,000 g for 10 minutes. Supernatant was saved for further fractions. The large membrane pellet was washed 2 times in 1X cell fractinaction buffer and resuspended in 100 μl laemmli buffer. For the vesicle and cytoplasmic fractions, the supernatant was cleaned by an additional spin at 7,000 g for 5 minutes and the pellet was discarded while the supernantant was centrifuged at 100,000 g for 1.5 hours and the vesicle pellet was resuspended in 100 μl laemmli buffer. For cytoplasmic fraction the supernantant was precipitated with 1 ml 60% Trichloroacetic acid and centrifuged at 15,000 g for 10 minutes to concentrate proteins. Pellet was washed once with acetone and resuspended in 100 μl laemmli loading dye. All samples were boiled for 20 minutes prior to loading on SDS-PAGE gel.

### Cilia isolation

Cilia were isolated as previously described [41,42]. IMCD3 cells were gown to confluency then serum starved for 2 days on 10 cm plates. Media was replaced with DMEM-F12 + 10% FCS. Cells were scraped off of plates in DMEM-F12 + 10% FCS. Remaining cells on plates were rinsed into centrifuge tube using PBS. Cells were pelleted at 1,000 g for 2 minutes. Cells were loosly resuspended once with PBS + 1 mM EDTA to remove free calcium and centrifuged at 1,000 g for 2 minutes. Cells were washed one more time in PBS to remove EDTA. Cells were resuspended in freshly prepared deciliation buffer (112 mM NaCl, 3.4 mM KCl, 10 mM CaCl_2_, 2.4 mM NaHCO_3_, 2 mM HEPES pH 7.4, 1 mM PMSF) and rocked for 15 minutes at 4°C to deciliate cells. Cell bodies were pelleted by centrifugation at 1,000 g for 5 minutes and the supernantant was cleared by centrifugation at 7,700 g for 5 minutes. The supernatant was added to the top of 45% (w/v) sucrose dissolved in de-ciliation buffer dyed with Bromophenol blue. The cilia fraction was centrifuged at 100,000 g for 1 hour. The interface band was collected and diluted 1:3 with deciliation buffer. Cilia were pelleted by centrifugation at 100000 g for 1 hour. The entire cilia pellet was loaded on SDS-PAGE gel for Western.

### Plasmids

pCS2-GFP-Daam1 and pCS2-GFP-Daam1(I698A) constructs were a gift from the Goode lab [33,34]. A mutation of A2822G was discovered in these plasmids, which resulted in the amino acid change of D941G. This mutation was corrected using site directed mutagenesis. The pCS2-GFP plasmid was generated by digesting pCS2-GFP-Cby1 (a gift from the Klymkowski lab) with ClaI–XbaI to remove the *cby1* gene [11]. The ends were blunted using phusion DNA polymerase (NEB) followed by ligation and transformation.

pCS2-mCherry-Daam1 and pCS2-mCherry-Daam1(I698A) constructs were generated by BamHI-NotI digestion of pCS2-GFP-Daam1 and pCS2-GFP-Daam1(I698A) and cloned into the same sites of pCS2-GFP-Cby1 replacing the GFP-Cby1 with Daam1. mCherry was PCR amplified from Cas9-mCherry (Addgene #78313) and cloned into the BamHI site to generate pCS2-mCherry-Daam1.

*sh-daam1* (#1 TTTCAGGAGATAGTATTGTGC, #2TAACATCAGAAATTCATAGCG, #3AAACAGGTCTTTAGCTTCTGC) and *sh-arhgef19* (TGCTTCTCACTTTCGGTCC) constructs were purchased from GE-Dharmacon. *sh-scrambled* plasmid was used as a negative control for 2D ciliation experiments. As *sh-daam1* (#1) construct is designed against the human *daam1*, and perfectly matches the *Canis lupis daam1* however, the target sequence is a two base pairs off of the mouse daam1 sequence. Therefore, a *sh-daam1* (*ms #1*) (TTTTAGGAGAAAGTATTGTGC) construct was generated to better target *daam1* in IMCD3 cells. The construct was made by cloning oligos containing a hairpin for the indicated sequence into EcoRI–AgeI sites of pLKO.1 as described in [56].

### Xenopus laevis

Wild type oocyte-positive *X*. *laevis* adults were purchased from Nasco (LM00531MX) and embryos were obtained from these adults and reared as previously described [66]. This protocol was approved by the University of Texas McGovern Medical School Institutional Animal Care and Use Committee (IACUC) (protocol #: AWC-16-0111). Microinjections were performed as previously described [38,67]. 10 nL of injection mix was injected into the indicated blastomere. For morpholino injections, 20 ng of Daam1 morpholino (5′ GCCGCAGGTCTGTCAGTTGCTTCTA 3′) [24,25] or Standard morpholino (5′ CCTCTTACCTCAGTTACAATTTATA 3′) was injected along with 500 pg of membrane targeted RFP RNA as a tracer to mark targeted cells into the V2-blastomere at the 8-cell stage to target the kidney [68].

### Data collection

All error bars in figures are reported as standard deviation across at least three independent trials. P-values were calculated using either Excel or Prism using a two tailed t-test. For nuclear counts, the image was grided off into 16 equal regions. Nuclei were counted in the same four tiles for all images. The average of the four tiles was obtained and used to estimate the total number of cells in the image. For cilia counts, all visible cilia were manually counted over an entire image (S5 Fig).

## Supporting information

S1 Fig Loss of Daam1 results in a reduction of primary cilia and mCherry-Daam1 localizes to vesicles carrying Ift88 in IMCD3 cells. Murine inner medullary collecting duct (IMCD3) were infected with either *sh-daam1* or a control construct then ciliated on glass coverslips. **A)** Cells were stained with acetylated α-Tubulin antibody (acTubulin) to label primary cilia (green), DAPI to label nuclei (blue), and phalloidin to label F-actin (magenta). Confocal imaging was used to analyze the effects Daam1 depletion upon primary ciliogenesis. Scale bars equal to 20 μm. B) Western blot of *sh-daam1* IMCD3 cell lysates showing depletion of Daam1 protein levels. GAPDH was used as a loading control. **C)** Cells were co-transfected with constructs that express mCherry-Daam1 and Ift88-GFP than imaged in live cells. Colocalization analysis was performed on individual cells using both Pierson and Manders formulas. Error bars are shown as ± SD and black dots indicate each image quatified. **D)** Representitive images of mCherry-Daam1 and Ift88-GFP in IMCD3 cells. Scale bars equal to 5 μm.
(TIF)

S2 Fig Phenotypes derived from control and *sh-daam1* knockdown. Daam1-depleted 3D MDCKII cyst were scored for the presence of (1) non-luminal cilia–cilia that do not protrude into central lumen, (2) multiple lumens and (3) hollow lumens-luminal clearance. Twenty cysts were randomly selected for analysis in three independent experiments. **A)** The graph indicates the relative percentage of cyst for each phenotype. Error bars are shown as ± SD; Significance was calculated using unpaired, two-tailed t-test; ns indicates p > 0.05, * indicates p < 0.05, **p < 0.01 **B)** Representative images of cysts with non-luminal cilia phenotype. In Daam1-depleted cysts, white arrows point at cilia protruding out into extracellular matrix. Scale bars equal to 10 μm.
(TIF)

S3 Fig Daam1 localiation at cilia and vesicles. **A-B)** Murine inner medullary collecting duct (IMCD3) cells were transfected with mCherry-Daam1 along with either Cby1-GFP or α-Tubulin-GFP. Cells were grown to confluency and serum starved to ciliate. Then cells were analyzed via confocal for colocalization of Daam1 and these ciliary markers. White boxes outline the ciliary transition zone in Cby images and cilia in α-Tubulin images. Scale bars equal to 10 μm. **C-D)** IMCD3 cells were ciliated fixed with glyoxal then stained for Ift88 and Daam1 using two diferent Daam1 antibodies. **E)** IMCD3 cells transfected with mCherry-Daam1 construct were grown to confluency and puncta were imaged using Airyscan super-resolution system. Vesicles are circled with a yellow dotted line.
(TIF)

S4 Fig Daam1-depletion does not lead to the absence of cilia during development of *Xenopus* embryonic kidneys. To further analyze the effect of Daam1 depletion on ciliogenesis, we fixed 8-cell Daam1 and Standard (control) morpholino injected embryos during early stages of kidney morphogenesis (stage 30). mRFP mRNA was used as a lineage tracer and coinjected with morpholinos. Stage 30-fixed embryos were immunostained with an antibody against anti-mRFP to visualize tracer (magenta) together with an Lhx1 antibody to label nephric progenitor cells (blue) and acetylated α-Tubulin antibody to label primary cilia (green). Subsequently, embryos were analyzed using a confocal laser-scanning microscope and representative maximum projections of Z-stack sections are shown. Acetylated α-Tubulin antibody stains primary cilia (white arrows), neurons (n) and multiciliated epidermal cells (mcc). Scale bar is equal to 50 μm.
(TIF)

S5 Fig Quantification methodology. **A)** To obtain unbiased quantitation of cell numbers in MDCKII depletion experiments (Figs 1 and 5), DAPI images were divided into a 4 x 4 grid. Nuclei were counted within the 4 indicated and the number of cells was averaged. This number was multiplied by 16 to obtain the approximate number of cells per image. **B)** Cilia labeled with using acetylated Lys40 tubulin antibody were counted manually. All cilia within an image were counted as shown in red. **C)** The lumen of 3D cysts were scored either for presence or absence of cilia. The lumens of cysts are marked with yellow dashed lines.
(TIF)
