# Supplementary figures and images for: Divergent roles of the Wnt/PCP Formin Daam1 in renal ciliogenesis
Source: PLoS One. 2019 Aug 30;14(8):e0221698. doi: 10.1371/journal.pone.0221698 (PMC6716777; doi:10.1371/journal.pone.0221698)

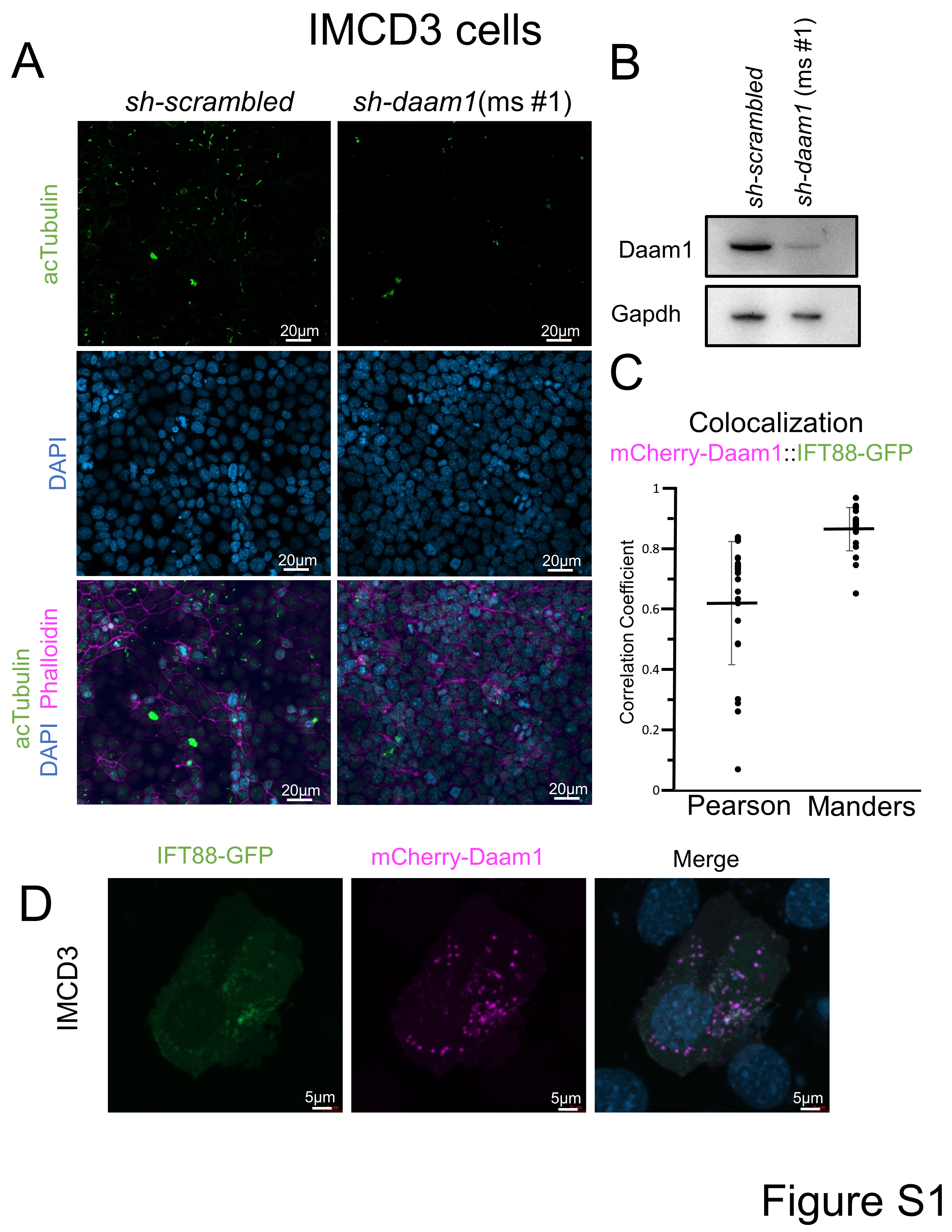

Supplement: S1 Fig — Murine inner medullary collecting duct (IMCD3) were infected with either sh-daam1 or a control construct then ciliated on glass coverslips. A) Cells were stained with acetylated α-Tubulin antibody (acTubulin) to label primary cilia (green), DAPI to label nuclei (blue), and phalloidin to label F-actin (magenta). Confocal imaging was used to analyze the effects Daam1 depletion upon primary ciliogenesis. Scale bars equal to 20 μm. B) Western blot of sh-daam1 IMCD3 cell lysates showing depletion of Daam1 protein levels. GAPDH was used as a loading control. C) Cells were co-transfected with constructs that express mCherry-Daam1 and Ift88-GFP than imaged in live cells. Colocalization analysis was performed on individual cells using both Pierson and Manders formulas. Error bars are shown as ± SD and black dots indicate each image quatified. D) Representitive images of mCherry-Daam1 and Ift88-GFP in IMCD3 cells. Scale bars equal to 5 μm. (TIF) [file pone.0221698.s001.TIF]

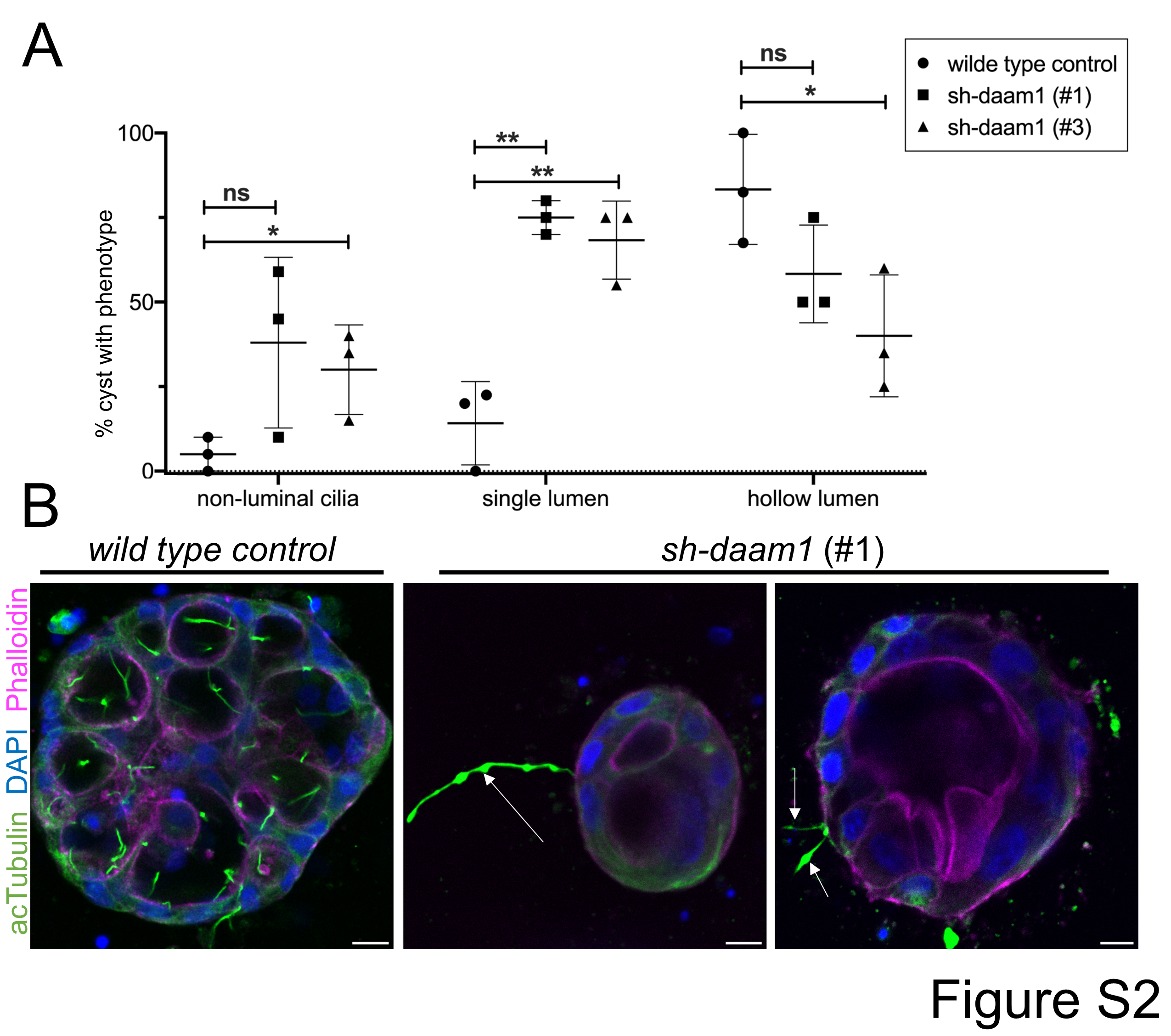

Supplement: S2 Fig — Daam1-depleted 3D MDCKII cyst were scored for the presence of (1) non-luminal cilia–cilia that do not protrude into central lumen, (2) multiple lumens and (3) hollow lumens-luminal clearance. Twenty cysts were randomly selected for analysis in three independent experiments. A) The graph indicates the relative percentage of cyst for each phenotype. Error bars are shown as ± SD; Significance was calculated using unpaired, two-tailed t-test; ns indicates p > 0.05, * indicates p < 0.05, **p < 0.01 B) Representative images of cysts with non-luminal cilia phenotype. In Daam1-depleted cysts, white arrows point at cilia protruding out into extracellular matrix. Scale bars equal to 10 μm. (TIF) [file pone.0221698.s002.TIF]

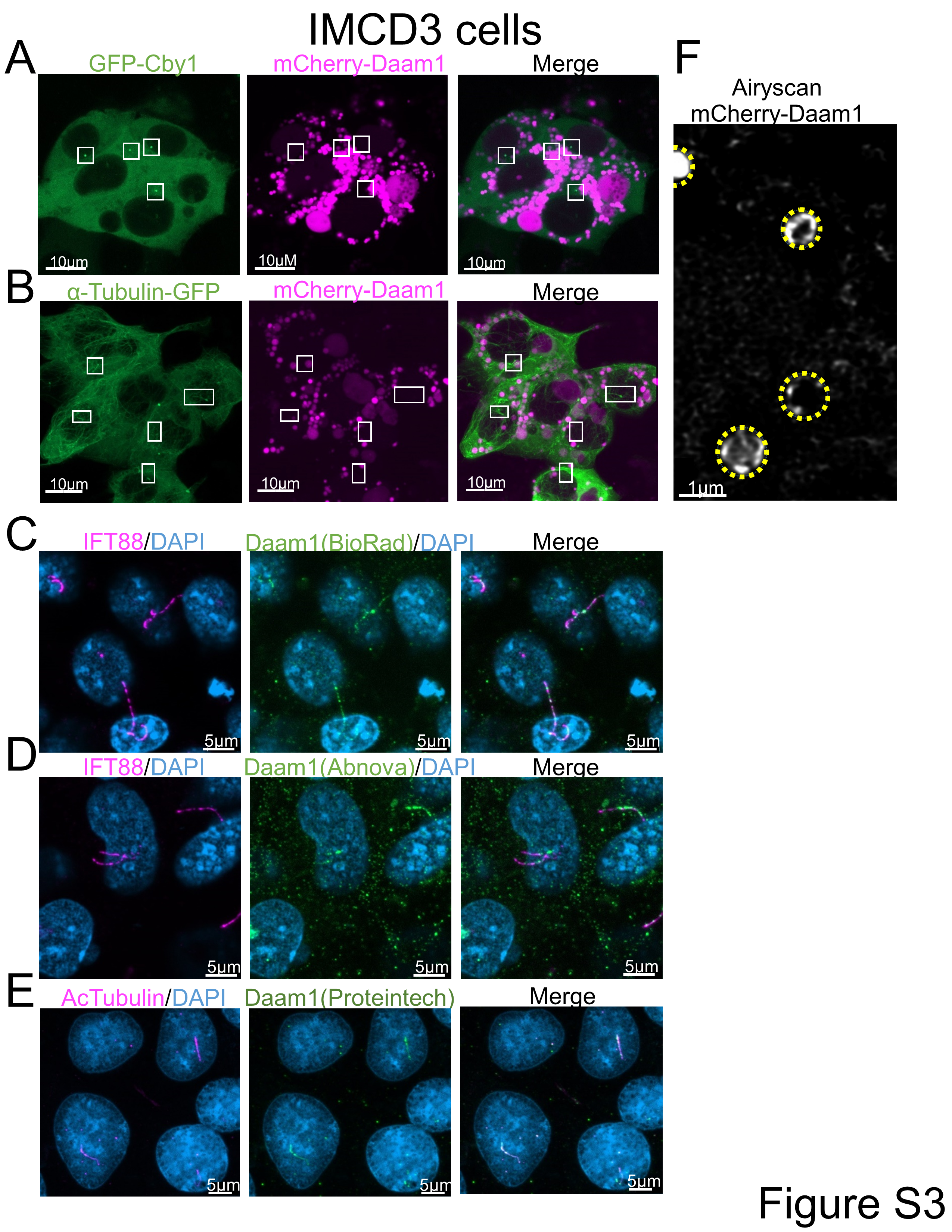

Supplement: S3 Fig — A-B) Murine inner medullary collecting duct (IMCD3) cells were transfected with mCherry-Daam1 along with either Cby1-GFP or α-Tubulin-GFP. Cells were grown to confluency and serum starved to ciliate. Then cells were analyzed via confocal for colocalization of Daam1 and these ciliary markers. White boxes outline the ciliary transition zone in Cby images and cilia in α-Tubulin images. Scale bars equal to 10 μm. C-D) IMCD3 cells were ciliated fixed with glyoxal then stained for Ift88 and Daam1 using two diferent Daam1 antibodies. E) IMCD3 cells transfected with mCherry-Daam1 construct were grown to confluency and puncta were imaged using Airyscan super-resolution system. Vesicles are circled with a yellow dotted line. (TIF) [file pone.0221698.s003.TIF]

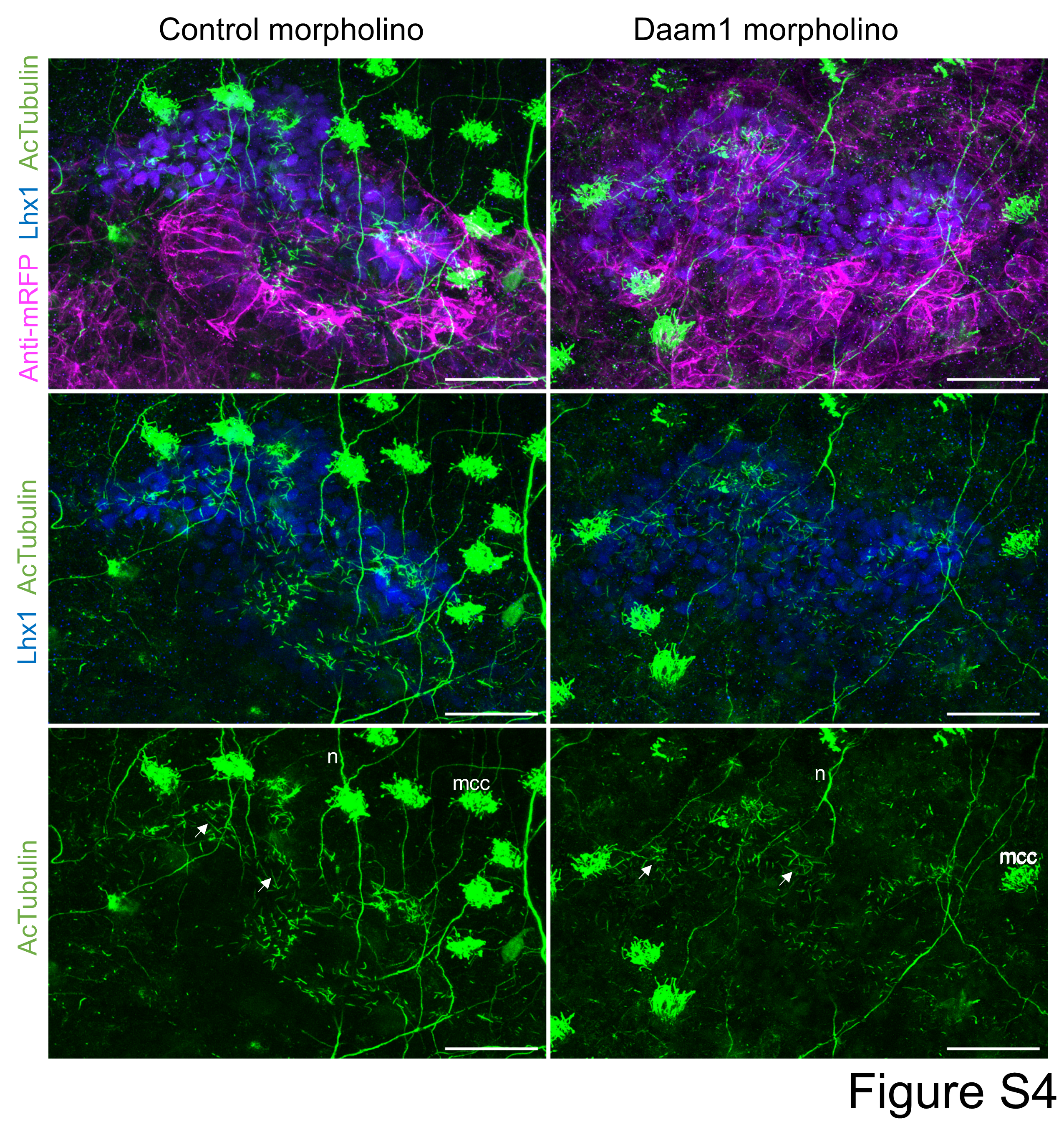

Supplement: S4 Fig — To further analyze the effect of Daam1 depletion on ciliogenesis, we fixed 8-cell Daam1 and Standard (control) morpholino injected embryos during early stages of kidney morphogenesis (stage 30). mRFP mRNA was used as a lineage tracer and coinjected with morpholinos. Stage 30-fixed embryos were immunostained with an antibody against anti-mRFP to visualize tracer (magenta) together with an Lhx1 antibody to label nephric progenitor cells (blue) and acetylated α-Tubulin antibody to label primary cilia (green). Subsequently, embryos were analyzed using a confocal laser-scanning microscope and representative maximum projections of Z-stack sections are shown. Acetylated α-Tubulin antibody stains primary cilia (white arrows), neurons (n) and multiciliated epidermal cells (mcc). Scale bar is equal to 50 μm. (TIF) [file pone.0221698.s004.TIF]

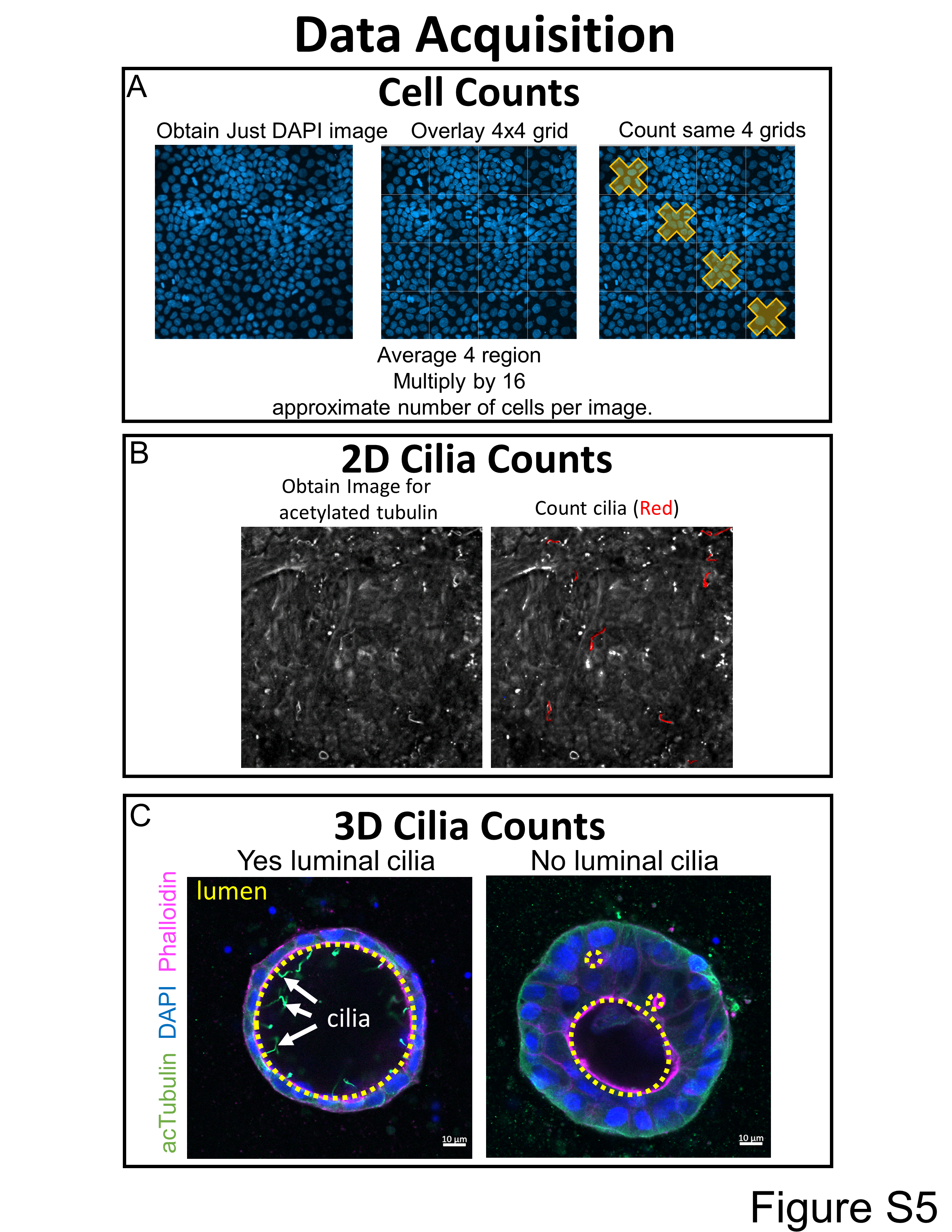

Supplement: S5 Fig — A) To obtain unbiased quantitation of cell numbers in MDCKII depletion experiments (Figs 1 and 5), DAPI images were divided into a 4 x 4 grid. Nuclei were counted within the 4 indicated and the number of cells was averaged. This number was multiplied by 16 to obtain the approximate number of cells per image. B) Cilia labeled with using acetylated Lys40 tubulin antibody were counted manually. All cilia within an image were counted as shown in red. C) The lumen of 3D cysts were scored either for presence or absence of cilia. The lumens of cysts are marked with yellow dashed lines. (TIF) [file pone.0221698.s005.TIF]
